# Parental Knowledge, Attitude, and Practice Toward Children’s Developmental Milestones in the Western Region of Saudi Arabia

**DOI:** 10.7759/cureus.52413

**Published:** 2024-01-16

**Authors:** Mohammed R. Alhayli, Ahmed A. Alhayli, Shatha M Alamri, Atheer M. Alamri, Jaber A Alfaifi, Mohsen H AlRashdi, Maryam H Almaqadi, Jawaher M Alamri, Abdulrahman R. Alhayli, Fahd Y. Somili

**Affiliations:** 1 Pediatrics, South Al-Qunfudah General Hospital, Ministry of Health, Al-Qunfudah, SAU; 2 Pediatrics, Al-Qunfudah College of Medicine, Umm Al-Qura University, Al-Qunfudah, SAU; 3 Pediatrics, University of Bisha, Bisha, SAU; 4 Pediatrics, Primary Health Care Center, Ministry of Health, Al-Qunfudah, SAU; 5 Pediatrics, King Fahd Central Hospital, Ministry of Health, Jazan, SAU

**Keywords:** awareness, cognitive, developmental milestones, practices, knowledge

## Abstract

Introduction: Parents' ability to engage and raise their children in a safe and appropriate manner is largely influenced by their knowledge of child development and childrearing. This study aimed to evaluate the parenting and developmental milestone (MS) knowledge of Western region Saudi parents and identify the related elements that influence their knowledge.

Methods: This cross‑sectional study was conducted for a period of six months. Ethical approval was duly sanctioned by the Institutional Review Board (IRB), and prior to participation, written informed consent was diligently procured from all the individuals involved in the study. In adherence to the paramount principles of privacy, rigorous measures were employed to de‑identify the personal data of the participants, thereby safeguarding their confidentiality and anonymity. All research procedures were meticulously executed in strict compliance with the pertinent guidelines and regulatory standards governing research ethics. The study cohort consisted of Saudi parents from the Western Province of Saudi Arabia who had children aged up to six years and expressed a genuine willingness to participate in the research. This commitment was reaffirmed through their informed consent. Notably, the inclusion criteria for parental involvement did not impose any restrictions based on age or ethnic origin, ensuring a diverse and inclusive representation of this crucial demographic group.

Results: For assessing parental awareness and knowledge about children's developmental MSs, we examined a diverse sample of 873 participants, predominantly comprising females (77.00%). The age distribution revealed that a substantial portion of the respondents were below 30 (37.00%). Most respondents (62.40%) sought information from medical physicians and pediatricians. Gender had a significant effect, with males showing a lower awareness level compared to females (Beta = -0.582, 95% confidence interval (CI) [-0.890, -0.274], p-value < 0.001). Marital status demonstrated significance, where divorced individuals showed a lower awareness level than widowed participants (Beta = -1.641, 95% CI [-2.993, -0.288], p-value = 0.017). At the same time, no significant differences were found for singles or married individuals.

Conclusion: Saudi parents lacked understanding of other parenting skills, such as a baby's personality and temperament, but they were well educated about some areas of childrearing, primarily physical safety precautions. It is advised that nurses and doctors give parenting advice to families at every step of their children's growth to educate and support them.

## Introduction

Parents' expectations of and interactions with their children are influenced by their understanding of and awareness of child development [1]. The literature has shown that a child's early life experiences play a significant role in shaping their future social skills, with the first five years of life being particularly critical for brain development [2].

Research conducted in industrialized nations revealed a significant correlation between a mother's capacity to improve her child's growth and her understanding of child development [3]. However, research indicates that parents who are ignorant about child development may overestimate their child's rate of development, which could result in unreasonable expectations, intolerance, and impatience [4].

A child's degree of achievement at a particular stage is indicated by the word "milestone" (MS). Because children develop at different rates, developmental MSs are not set in stone and have a typical range of variation. Developmental MSs serve as approximate indicators of when one can anticipate specific changes in a child's growth and development as they progress through different stages of childhood. Nevertheless, it is important to acknowledge the inherent variability in individual development, making it a challenging task to predict when a child will master a particular skill precisely [5].

Understanding early life MSs, continuous developmental processes, and being familiar with parenting techniques are all ways to gain knowledge about child development. Research from several industrialized nations revealed a significant correlation between mothers' increased capacity to support their children's development and their understanding of child development [6].

The ongoing process of developing and using the information and abilities necessary for organizing, conceiving, giving birth, raising, and taking care of children is referred to as "parenting" [6]. Proficient in their child's development, parents exhibit high levels of efficacy and competence in parenting. Conversely, even though they were effective parents, parents with insufficient information had insufficient parenting skills [7,8].

In the 1980s and 1990s of the previous century, child psychologists were concerned with developmental expectations and mothers' awareness of developmental MSs. This is why it is a common topic in Western literature [9]. Studies from the Arab world, Saudi Arabia included, are, on the other hand, rare. Thus, the purpose of this study was to evaluate Western Saudi parents' knowledge, attitudes, and practices regarding developmental MSs for infants, as well as the sociodemographic factors relevant to learning and the sources of information regarding these MSs.

## Materials and methods

This cross‑sectional study and an online survey were sent to participants through a short message service (SMS). They explained the research and its purpose, with informed consent required before participating and completing the questionnaire. This study’s protocol with IRB number HAPO-02-K-012-2023-05-1628 was approved by the Institutional Review Board (IRB) of Umm Al-Qura University. Data de‑identification measures were employed to safeguard participant privacy, and research procedures adhered to relevant guidelines and regulations. The study included Saudi parents from the Western Province with children up to six years who provided informed consent. No age or ethnic origin restrictions were imposed. Exclusions comprised parents of infants with confirmed developmental abnormalities, complex perinatal and postnatal histories, and neurologically deficient infants, as these factors could skew developmental norm findings. Participant recruitment occurred through visits to Governmental Primary Healthcare Centers (PHCs) in the Western Province of Saudi Arabia. The total sample size was 873 using the purposive sampling technique during the study period.

Methodology

Data collection was executed through a questionnaire comprising three distinct sections. The initial section was designed to capture sociodemographic information, while the second section involved the administration of "The Knowledge of Infant Development Inventory (KIDI)," an instrument developed by David McPhee [10].

Before applying the KIDI tool in this study, formal permission to utilize it was sought and obtained from the original author. The KIDI tool, structured as a univariate scale consisting of 58 items, was specifically devised to evaluate an individual's knowledge pertaining to parenting practices and child developmental processes. The first 39 items of the original KIDI focused on assessing knowledge regarding normative child behaviors, requiring respondents to express their agreement, disagreement, or uncertainty regarding various statements. In the subsequent part (items 40-58), which centered on a child's developmental MSs, respondents were prompted to specify whether a younger or older child could attain a given developmental MS when expressing disagreement [10].

The KIDI instrument encompassed two segments, one dedicated to childrearing practices and the other to developmental MSs. The third section of the questionnaire was a modified adaptation of the "Parental Sources of Information Questionnaire," which had been employed in a previous, similar study conducted in Jordan [6]. Approval from the original authors was secured prior to incorporating this questionnaire into the present study.

Statistical analysis

Statistical analysis was performed using IBM SPSS Statistics for Windows, Version 26 (Released 2019; IBM Corp., Armonk, New York, United States). Categorical sociodemographic data were expressed in terms of frequencies and percentages. Mann-Whitney and Kruskal-Wallis tests were employed to assess the relationship between the outcome numerical variable and sociodemographic factors. These tests provided medians, interquartile ranges, and associated p-values.

Generalized linear regression models were established to ascertain the predictors of high scores in parental awareness and knowledge concerning children's developmental MSs. These models were based on the sociodemographic variables that exhibited statistical significance in relation to the primary outcome. The results of the regression analyses were presented in the form of beta coefficients, along with their corresponding 95% CIs. Statistical significance was determined at a p-value of less than 0.05.

## Results

As per Table 1, in our study on parental awareness and knowledge about children's developmental MSs, we examined a diverse sample of 873 participants, predominantly female (77.00%). The data in Table 1 has been represented as N (number of participants) and % (percentage). A p-value of less than 0.05 was considered statistically significant.

**Table 1:** Sociodemographic data (n = 873) Sociodemographic data (n = 873) representing the number of participants (N) and percentage (%) in the study. Significance level: p<0.05.

	Category	N	%
Gender	Male	201	23.00%
	Female	672	77.00%
Age	< 30	323	37.00%
	31-39	235	26.90%
	40-49	226	25.90%
	50-59	77	8.80%
	60 or more	12	1.40%
Marital status	Single	198	22.70%
	Married	635	72.70%
	Divorced	29	3.30%
	Widowed	11	1.30%
Nationality	Saudi	772	88.40%
	Non-Saudi	101	11.60%
Geographical region	Northern	0	0.00%
	Southern	97	11.10%
	Central	39	4.50%
	Eastern	51	5.80%
	Western	686	78.60%
Residency	Urban area	700	80.20%
	Rural area	173	19.80%
Educational level	Uneducated	6	0.70%
	Elementary	12	1.40%
	Middle	29	3.30%
	High school	196	22.50%
	University	582	66.70%
	Postgraduate	48	5.50%
Employment	Student	138	15.80%
	Unemployed	287	32.90%
	Employed	397	45.50%
	Entrepreneur	12	1.40%
	Retired	39	4.50%
Monthly income	Less than 5000	398	45.60%
	5000-10000	275	31.50%
	More than 10000	200	22.90%

The age distribution revealed that a substantial portion of the respondents were below 30 (37.00%). Regarding marital status, the majority were married (72.70%). A significant proportion of the participants identified as Saudi nationals (88.40%). Geographically, the majority resided in the Western region (78.60%). In terms of residency, 80.20% lived in urban areas. Educational levels varied, with a significant proportion holding university degrees (66.70%). Employment status showed diversity, with 45.50% being employed. The monthly income distribution varies, with the highest proportion (45.60%) earning less than 5000.

Based on Table 2, the results of our study on parental awareness of children's developmental MSs revealed significant variation in participants' perceptions of when infants typically reach various developmental stages. The data in Table 2 has been represented as N (number of participants) and % (percentage). A p-value of less than 0.05 was considered statistically significant. For the question regarding when an infant starts to move their head, the majority (33.80%) believed it occurs at one week. When it comes to infants turning over, 76.60% of respondents believed this occurs at six months. In the case of standing, the most common perception was that it begins at 10 months (54.30%). Concerning walking alone steadily, 38.60% believed it happens at 12 months. Climbing stairs was thought to start at 24 months by 44.30% of respondents. The ability to follow things with the eyes was perceived by 59.50% to start at six weeks. Most participants believe a child approaches objects at six months (49.70%). A substantial 77.10% believed this begins at three years and above for manual skills like drawing.

**Table 2:** Descriptive analysis of parental awareness and knowledge about children's developmental milestone (gross and fine motors) (n = 873) Descriptive analysis of parental awareness and knowledge about children's developmental milestones (gross and fine motors) (n = 873), representing the number of participants (N) and percentage (%) in the study. Significance level: p<0.05.

Parameter	Category	N	%
When does the infant start to move his head?	1 week	295	33.80%
	3 weeks	263	30.10%
	6 weeks	156	17.90%
	1 month	159	18.20%
When does the infant begin to turn over?	1 month	120	13.70%
	6 months	669	76.60%
	10 months	65	7.40%
	1 year	19	2.20%
When does the infant start to stand?	8 months	233	26.70%
	10 months	474	54.30%
	1 year and half	150	17.20%
	2 years	16	1.80%
When does the child walk alone steadily?	10 months	65	7.40%
	12 months	337	38.60%
	15 months	295	33.80%
	20 months	176	20.20%
When does the child start to climb stairs?	12 months	91	10.40%
	14 months	121	13.90%
	18 months	274	31.40%
	24 months	387	44.30%
When can a child follow things with his eyes?	At birth	41	4.70%
	1 week	214	24.50%
	6 weeks	519	59.50%
	1 year	99	11.30%
When can a child approach an object?	1 week	14	1.60%
	1 month	47	5.40%
	6 months	434	49.70%
	1 year	378	43.30%
When can a child do manual skills like drawing?	1 month	18	2.10%
	6 months	32	3.70%
	1 year	150	17.20%
	At 3 years and above	673	77.10%

Respondents reported diverse beliefs about when a child begins to respond to the mother's voice, with 30.50% perceiving this to occur at three weeks. The initiation of saying "Mama and Papa" was thought to commence predominantly at 10 months (36.00%). The MS of smiling was believed to start at six weeks by (54.50%) of participants, while the recognition of age and name was perceived as beginning mostly at two years (31.00%). Saying "bye-bye" was thought to start at one year by 41.40%, and drinking from a cup was perceived as achievable at 18 months by 33.20%. Finally, regarding the control of urination, a significant 48.20% believed it begins at three years (Table 3).

**Table 3:** Descriptive analysis of parental awareness and knowledge about children's developmental milestone (speech and language, social and emotional) (n = 873) Descriptive analysis of parental awareness and knowledge about children's developmental milestone (speech and language, social and emotional) (n = 873), representing the number of participants (N) and percentage (%) in the study. Significance level: p<0.05.

Parameter	Category	N	%
At what age does the child begin to respond to the mother's voice?	1 week	199	22.80%
	3 weeks	266	30.50%
	6 weeks	231	26.50%
	1 month	177	20.30%
At what age does a child start saying "Mama and Papa"?	4 months	73	8.40%
	6 months	188	21.50%
	8 months	298	34.10%
	10 months	314	36.00%
At what age does a child start smiling?	6 weeks	476	54.50%
	8 weeks	212	24.30%
	12 weeks	98	11.20%
	14 weeks	66	7.60%
	1 month	8	0.90%
	2 months	13	1.50%
At what age does a child begin to know his age and his name?	1 year	244	27.90%
	2 years	271	31.00%
	3 years	240	27.50%
	4 years	118	13.50%
At what age does a child start saying "bye-bye"?	6 months	111	12.70%
	10 months	242	27.70%
	1 year	361	41.40%
	2 years	159	18.20%
At what age can a child drink from a cup?	12 months	282	32.30%
	18 months	290	33.20%
	2 years	234	26.80%
	3 years	67	7.70%
At what age does a child begin to control urination?	1 year	46	5.30%
	2 years	175	20.00%
	3 years	421	48.20%
	4 years	231	26.50%

**Figure 1:**
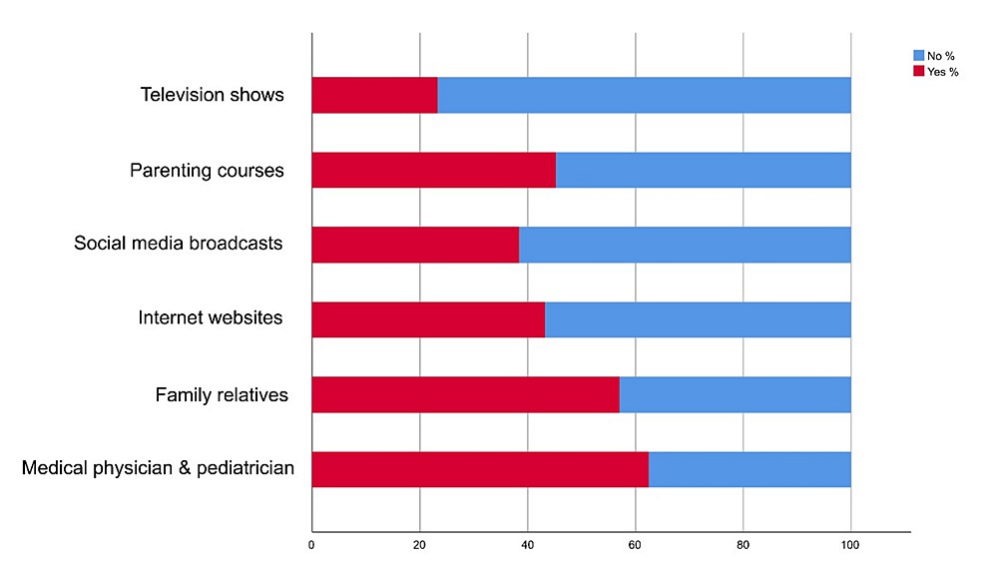
Source of information regarding parental awareness and knowledge about children's developmental milestone The data has been represented as %.

Most respondents (62.40%) sought information from medical physicians and pediatricians (Figure 1).

The overall median and IQR of knowledge score is 6.0 (5.0-7.0). Regarding Table 4, a statistically significant difference (p-value < 0.001) was observed between genders, with females reporting a slightly higher median awareness score (6.0, IQR 5.0-7.0) compared to males (5.0, IQR 4.0-7.0). Age also showed significant differences (p-value < 0.001), where participants aged below 30 and between 31-39 and 40-49 had a median awareness score of 6.0, whereas those aged 50-59 had a median of 6.0 (IQR 4.5-7.0), and those aged 60 or more had a slightly lower median of 5.5 (IQR 3.25-7.0). Marital status significantly impacted (p-value = 0.007) awareness, with divorced individuals reporting a median of 5.0 (IQR 4.0-6.5), while singles, married, and widowed participants had a median score of 6.0. The geographical region demonstrated a significant difference (p-value = 0.044), with the Southern region showing a median awareness score of 5.0 (IQR 3.0-7.0), while other regions had a median of 6.0. However, no significant differences were found based on nationality, residency, educational level, employment status, or monthly income.

**Table 4:** Association between sociodemographic data and parental awareness and knowledge about children's developmental milestone Descriptive analysis of knowledge scores with medians (median) and interquartile ranges (IQR), along with p-values for various demographic parameters (n = 873). Significance level: p < 0.05.

Parameter	Category	Median (IQR)	p-value
Gender	Male	5.0 (4.0-7.0)	< 0.001
	Female	6.0 (5.0-7.0)	
Age	< 30	6.0 (4.0-7.0)	< 0.001
	31-39	6.0 (5.0-7.0)	
	40-49	6.0 (5.0-7.0)	
	50-59	6.0 (4.5-7.0)	
	60 or more	5.5 (3.25-7.0)	
Marital status	Single	6.0 (4.0-7.0)	0.007
	Married	6.0 (5.0-7.0)	
	Divorced	5.0 (4.0-6.5)	
	Widowed	6.0 (6.0-8.0)	
Nationality	Saudi	6.0 (5.0-7.0)	0.768
	Non-Saudi	6.0 (4.0-7.0)	
Geographical region	Southern	5.0 (3.0-7.0)	0.044
	Central	6.0 (5.0-7.0)	
	Eastern	6.0 (5.0-7.0)	
	Western	6.0 (5.0-7.0)	
Residency	Urban area	6.0 (5.0-7.0)	0.599
	Rural area	6.0 (4.0-7.0)	
Educational level	Uneducated	2.5 (0.75-6.75)	0.109
	Elementary	5.0 (4.0-6.75)	
	Middle	6.0 (3.0-7.0)	
	High school	6.0 (5.0-7.0)	
	University	6.0 (5.0-7.0)	
	Postgraduate	6.0 (5.0-8.0)	
Employment	Student	6.0 (4.0-7.0)	0.079
	Unemployed	6.0 (5.0-7.0)	
	Employed	6.0 (5.0-7.0)	
	Entrepreneur	5.5 (4.25-7.0)	
	Retired	6.0 (4.0-7.0)	
Monthly income	Less than 5000	6.0 (5.0-7.0)	0.153
	5000-10000	6.0 (4.0-7.0)	
	More than 10000	6.0 (5.0-7.0)	

In Table 5, the results of our regression analysis examining the impact of various demographic factors on parental awareness of children's developmental MSs revealed several notable findings. Gender had a significant effect, with males showing a lower awareness level compared to females (Beta = -0.582, 95% CI [-0.890, -0.274], p-value < 0.001). Marital status demonstrated significance, where divorced individuals showed a lower awareness level than widowed participants (Beta = -1.641, 95% CI [-2.993, -0.288], p-value = 0.017). At the same time, no significant differences were found for singles or married individuals.

**Table 5:** Linear regression showing predictors of parental awareness and knowledge about children's developmental milestone based on the statistically significant sociodemographic data Regression analysis results with Beta values, 95% confidence intervals (CI), and p-values for the impact of various demographic factors on parental awareness of children's developmental milestones (n = 873). Significance level: p < 0.05.

Parameter	Category	Beta	95% CI		p-value
			LB	UB	
Gender	Male	-0.582	-0.890	-0.274	< 0.001
	Female	Ref.	Ref.	Ref.	Ref.
Age	< 30	0.230	-0.916	1.377	0.693
	31-39	0.968	-0.168	2.103	0.095
	40-49	0.878	-0.255	2.011	0.129
	50-59	0.352	-0.821	1.525	0.556
	60 or more	Ref.	Ref.	Ref.	Ref.
Marital status	Single	-0.863	-2.080	0.355	0.165
	Married	-0.762	-1.935	0.412	0.203
	Divorced	-1.641	-2.993	-0.288	0.017
	Widowed	Ref.	Ref.	Ref.	Ref.
Geographical region	Southern	-0.234	-0.653	0.186	0.275
	Central	0.001	-0.613	0.616	0.996
	Eastern	-0.393	-0.932	0.147	0.153
	Western	Ref.	Ref.	Ref.	Ref.

## Discussion

The current study outlines Western Saudi Arabia's parents' understanding of newborn development stages and childrearing. The well-being of children, parents, and society as a whole depends on parenting expertise and a grasp of childrearing and development processes. As far as we know, Saudi Arabia lacks such information in the Western region.

In contrast, Saudi women demonstrated a greater understanding of the parent-child relationship [6]. As opposed to 25% in the previous study, 42.4% of our study participants reject the notion that carrying a crying infant will spoil him or her [6]. In a similar vein, our study revealed a more positive interpretation of the causes of a baby's crying than the Jordanian one [6], with 51.7% of the moms in our sample disagreeing that babies cry merely to cause problems compared to 29.5% of the mothers in the Jordanian study.

Typically, parents get virtually exclusively physical information from healthcare practitioners, with little to no emphasis placed on cognitive, emotional, and parent-infant interaction skills [11]. Furthermore, it is evident that the primary focus of physical healthcare services is on vaccines, physical examinations, and evaluations of the growth and development of infants. According to earlier research in the literature, pediatricians have a crucial role in parenting practices by focusing on basic treatment and health maintenance while ignoring discussions with them regarding parts of childrearing education [12]. The current investigation revealed a high degree of physical aspect understanding.

Most services provided by PHCs concentrate on immunizations, growth parameter checks, and general health-related concerns like constipation, feeding difficulties, and nutritional challenges. This highlights our population's general lack of maternal information. Because PHC practitioners handle such general health-related themes, the health and safety subscale, which had the greatest level of knowledge (63.4%) among mothers, is an excellent example of such a shortage in the PHC function. Another study conducted by Arabs has likewise made the same observation. Furthermore, fundamental security and physical health subjects like immunization and diet are periodically covered in-depth on various media channels. While most pediatricians address fundamental health maintenance issues, worldwide studies have revealed that other areas of childrearing and development instruction are often disregarded [13,14].

The primary sources of information regarding developmental MSs in the current study were medical education websites, mothers' families, healthcare providers, and the presence of an older kid. The current way information is accessed is expected to change as the internet becomes more widely available [15]. Parents also depend on information from their own families, and studies have shown that mothers' understanding of infant development is closely correlated with that of their own mothers [15]. Contrary to our findings [16], a similar Iraqi study found that mothers learned the majority of knowledge about their children's developmental MSs from their own experiences (71.5%), with very small percentages learning it from doctors (16.5%) and PHC institutions (5.5%).

According to other research of a similar nature, moms who are employed and educated - especially those with a higher degree of education - have a good developmental understanding [15]. The results of the current study indicated a substantial correlation between the mother's work and understanding of the overall development MSs. This went against the findings [16], which showed a strong correlation between homemaker moms' understanding of motor development and their level of education. Regarding parity, the number of delivered kids and the mother's awareness of a child's developmental MSs did not significantly correlate, consistent with a 2015 study that also revealed no significant correlation [17].

## Conclusions

A child's early years significantly impact their future social, physical, mental, and cognitive development. Furthermore, as parents are typically in charge of providing childcare at this crucial time in most cultures and groups, their understanding of developmental MSs affects the type and standard of care the kid receives. However, parents have gaps in their knowledge of children's development, even though women are better at identifying MSs than men. The majority of parents lacked appropriate levels of knowledge and were unable to accurately respond to questions about the developmental MSs of their children.

In general, it is believed that Western region Saudi parents only have a limited understanding of certain areas of childrearing - primarily physical safety precautions. It was noted that there was a lack of expertise in other parenting abilities. The lack of emphasis on interactions between mothers and healthcare professionals may be the primary cause of this inadequate knowledge, particularly given that mothers mainly acquired their childrearing knowledge from nurses and physicians. Nurses and doctors are advised to educate and assist families by providing parenting education across all developmental stages of children as a workable solution to such an issue.
